# Editorial: Responsible Robotics

**DOI:** 10.3389/frobt.2022.937612

**Published:** 2022-06-21

**Authors:** Martim Brandão, Masoumeh Mansouri, Martin Magnusson

**Affiliations:** ^1^ Department of Informatics, King’s College London, London, United Kingdom; ^2^ School of Computer Science, University of Birmingham, Birmingham, United Kingdom; ^3^ School of Science and Technology (AASS), Örebro University, Örebro, Sweden

**Keywords:** robotics, responsible innovation, responsible robotics, trustworthy robotics, critical robotics, AI and society, robot ethics

## 1 Responsible AI and Robotics

Recent work in both academia, industry, and journalism has brought widespread attention to various kinds of harmful impact that AI can have on society. These are very often concentrated on marginalized social groups. AI algorithms may unintentionally reinforce social prejudice Bolukbasi et al. (2016) and biased conceptions of gender Adams and Loideáin (2019); Hamidi et al. (2018), race Sweeney (2013), age Rosales and Fernández-Ardávol (2019) or disabilities Guo et al. (2020), they may lead to unfair access to opportunities Dastin (2018); Angwin et al. (2016), discriminatory pricing practices Bar-Gill (2019); Hannak et al. (2014), etc. Recent work has also shown that many seemingly technical issues in machine learning are actually socio-technical. For example: the over-fitting of machine learning models, the choice of dataset or learning objective, and other aspects of learning may lead to algorithms performing poorly on unrepresented or unmodeled groups of people Brandao (2019); Barocas et al. (2019); Buolamwini and Gebru (2018). A growing community of Fairness, Accountability, Transparency, and Ethics of AI^1^ is now approaching these Research Topic from a socio-technical point-of-view, in order to identify, understand, and alleviate such issues.

Robotics, as a technology focused on automation and intelligent behavior, also abounds in similar ethical and social issues that need to be identified, characterized, and considered in design. While many of the same problems with AI will also be present in robotics, the physical nature of robotics raises new aspects of the social and ethical nature of these technologies. As one example: models that are considerably less accurate on certain groups of people can lead to physical safety differentials Brandao (2019), where robots or autonomous vehicles using those models are more likely to collide with those groups. Additionally, there are physical safety concerns with respect to surgical and other medical robots Yang et al. (2017); Ficuciello et al. (2019), as well as concerns of physical and political security—not least concerning autonomous weapon systems and the dual-use of robot technologies like autonomous cars and drones Brundage et al. (2018); Sparrow (2007).

The physical design and visual appearance of robots also introduce new aspects to responsible development. For example, people’s moral evaluation of robot decisions can be affected by whether the robot is more or less human-like Malle and Scheutz (2016), the design of robots in a care setting affects caregivers and caretakers van Wynsberghe (2021); Kubota et al. (2021), the choice of sensors, measurements and motion has an impact of privacy Calo (2011); Eick and Antón (2020); Luo et al. (2020), and the ethics of deception takes on new shape Danaher (2020).

The robotics community has been discussing ethics for long^2^. Recent workshops have also started bringing attention to philosophical problems in robotics^3^ and issues such as bias^4^ and transparency^5^. These efforts share a common goal of developing robotics technologies responsibly—they are part of “Responsible Robotics” or “Trustworthy Robotics.”

A similar effort on “Critical Robotics” Serholt et al. (2021) has focused on questioning current practices in robotics research. These range from how older adults are represented in HRI Burema (2021) and ethical issues in education robots Serholt et al. (2017), to normative dimensions of speech used by researchers Brandao (2021), their technological optimism Šabanović (2010) and the influence of their social background in research directions Forsythe (2001); Šabanović (2010).

## 2 This Research Topic

This Research Topic gathers a diverse set of articles on Responsible Robotics. They range from user studies and philosophical inquiry, to modeling, algorithmic, and governance methods. Our goal when organizing this Research Topic was exactly to join various approaches in a single edition—to allow for greater multidisciplinary exchange under the common mission of Responsible Robotics. We believe that Responsible Robotics should focus both on *identifying* social and ethical issues, and on *designing* methods to account for (and alleviate) such issues—thus the focus of this edition on both understanding and *acting* on social and ethical issues.

Two articles in the Research Topic are focused on eliciting social and ethical issues *from users and stakeholders*. Lutz and Tamò-Larrieux investigate privacy concerns of lay users and their impact on technology use intentions, when using social robots that are either privacy-friendly or privacy-invasive (e.g., listen to conversations, share data with third parties). Colombino et al. use ethnographic studies, interviews and futuristic autobiographies to identify organizational principles, potential roles, and ethical design considerations for a robot that collaborates with disabled employees.

Three articles are more focused on methods, or socio-technical solutions to ethical problems in robotics. Webb et al., for example, focus on methods for conducting investigations of accidents involving humans and robots. In particular, they propose and preliminarily evaluate a role-play-based methodology for investigating accidents, and to evaluate the testimonies that humans can give in forensic investigations of such accidents. Hurtado et al. focus on issues of harmful social bias in robot learning and how they could be detected and alleviated. Namely, they show through various examples how social robot navigation techniques that mimic human behavior may lead to harmful behavior, such as higher intrusion of personal space or longer waiting times for some groups compared to others. Winfield et al. focus on issues of transparency from a governance perspective. They describe a new draft standard on transparency for autonomous systems, with several contributions such as transparency levels, measurability, stakeholders, and example-based guidance on using the draft standard.

We then dive into philosophical inquiry and frameworks for robot ethics. Rhim et al. combine work in moral philosophy and psychology to propose a model that explains human decision-making in moral dilemmas involving autonomous vehicles. Pirni et al. consider aspects of autonomy and vulnerability in the ethics of designing care robots. And Kuipers argues that AI and robotics technologies rely heavily on over-simplified models, and that the widespread use of such models can lead to the erosion of trust and cooperation effectiveness. The article can serve as an argument for why more attention should be given to the *modeling* of complex socio-technical factors in AI/robotics.

Finally, two articles in the Research Topic dive into issues of jobs and economics in robotics and automation. Studley argues that we should consider how robotics impacts global supply chains, international development, and global economic disparities. Kyvik Nordås and Klügl then use modeling to understand the uptake of automation technologies and its relationship with unemployment and engineering, consultancy, and manufacturing jobs. The authors use this analysis to suggest an automation policy focus on user costs and education.

We believe that the contributions collected in this Research Topic can be relevant to roboticists, AI practitioners, policy makers and any other stakeholders concerned with the societal impacts of AI and robotics. We hope this Research Topic will stimulate future work on responsible robotics.

We end with an important remark. While the abundance of social and ethical issues raised in this editorial and this Research Topic might feel overwhelming or hopeless, we believe the opposite is the case. Responsible Robotics is about clearly identifying potential issues, because by doing so it is also possible to work towards responsible methods that mitigate them. This ultimately facilitates the application of robotics and AI in ways that increase safety, efficiency, and wellbeing in many areas of life: transportation, healthcare, work life, just to name a few.

## Footnotes

1 Example venues: ACM Conference on Fairness, Accountability, and Transparency (FAccT), AAAI/ACM Conference on Artificial Intelligence, Ethics, and Society (AIES).

2 ICRA 2007/2009/2011 workshops on Roboethics, ICRA 2014 workshop on “Robotics and Military Applications”.

3 Robophilosophy Conference.

4 ICRA 2019 workshops on “Bias-sensitizing robot behaviours” and “Unlearning biases in robot design”.

5 HRI 2022 workshop on “Fairness and Transparency in HRI,” ICRA 2020 workshop “Against robot dystopias”.
